# Patient characteristics, health seeking and delays among new sputum smear positive TB patients identified through active case finding when compared to passive case finding in India

**DOI:** 10.1371/journal.pone.0213345

**Published:** 2019-03-13

**Authors:** Hemant Deepak Shewade, Vivek Gupta, Srinath Satyanarayana, Prabhat Pandey, U. N. Bajpai, Jaya Prasad Tripathy, Soundappan Kathirvel, Sripriya Pandurangan, Subrat Mohanty, Vaibhav Haribhau Ghule, Karuna D. Sagili, Banuru Muralidhara Prasad, Sudhi Nath, Priyanka Singh, Kamlesh Singh, Ramesh Singh, Gurukartick Jayaraman, P. Rajeswaran, Binod Kumar Srivastava, Moumita Biswas, Gayadhar Mallick, Om Prakash Bera, K. N. Sahai, Lakshmi Murali, Sanjeev Kamble, Madhav Deshpande, Naresh Kumar, Sunil Kumar, A. James Jeyakumar Jaisingh, Ali Jafar Naqvi, Prafulla Verma, Mohammed Salauddin Ansari, Prafulla C. Mishra, G Sumesh, Sanjeeb Barik, Vijesh Mathew, Manas Ranjan Singh Lohar, Chandrashekhar S. Gaurkhede, Ganesh Parate, Sharifa Yasin Bale, Ishwar Koli, Ashwin Kumar Bharadwaj, G. Venkatraman, K. Sathiyanarayanan, Jinesh Lal, Ashwini Kumar Sharma, Raghuram Rao, Ajay M. V. Kumar, Sarabjit Singh Chadha

**Affiliations:** 1 International Union Against Tuberculosis and Lung Disease (The Union), South-East Asia Office, New Delhi, India; 2 International Union Against Tuberculosis and Lung Disease (The Union), Paris, France; 3 All India Institute of Medical Sciences (AIIMS), New Delhi, India; 4 Voluntary Health Association of India (VHAI), New Delhi, India; 5 Post Graduate Institute of Medical Education and Research (PGIMER), Chandigarh, India; 6 MAMTA Health Institute for Mother and Child, New Delhi, India; 7 Catholic Health Association of India (CHAI), Telangana, India; 8 Resource Group for Education & Advocacy for Community Health (REACH), Chennai, India; 9 Population Services International (PSI), New Delhi, India; 10 State TB Cell, Department of Health & Family Welfare, Government of Bihar, Patna, India; 11 State TB Cell, Department of Health & Family Welfare, Government of Tamil Nadu, Chennai, India; 12 State TB Cell, Health Department, Government of Maharashtra, Pune, India; 13 State TB Cell, Department of Health & Family Welfare, Government of Chattisgarh, Raipur, India; 14 State TB Cell, Department of Health & Family Welfare, Government of Punjab, Chandigarh, India; 15 State TB Cell, Department of Health & Family Welfare, Government of Kerala, Thiruvananthapuram, India; 16 Catholic Bishops’ Conference of India-Coalition for AIDS and Related Diseases (CBCI-CARD), New Delhi, India; 17 Emmanuel Hospital Association (EHA), New Delhi, India; 18 Central TB Division, Revised National Tuberculosis Control Programme, Ministry of Health and Family Welfare, Government of India, New Delhi, India; Imperial College London, UNITED KINGDOM

## Abstract

**Background:**

*Axshya SAMVAD* is an active tuberculosis (TB) case finding (ACF) strategy under project *Axshya* (*Axshya* meaning ‘free of TB’ and *SAMVAD* meaning ‘conversation’) among marginalized and vulnerable populations in 285 districts of India.

**Objectives:**

To compare patient characteristics, health seeking, delays in diagnosis and treatment initiation among new sputum smear positive TB patients detected through ACF and passive case finding (PCF) under the national TB programme in marginalized and vulnerable populations between March 2016 and February 2017.

**Methods:**

This observational analytic study was conducted in 18 randomly sampled *Axshya* districts. We enrolled all TB patients detected through ACF and an equal number of randomly selected patients detected through PCF in the same settings. Data on patient characteristics, health seeking and delays were collected through record review and patient interviews (at their residence). Delays included patient level delay (from eligibility for sputum examination to first contact with any health care provider (HCP)), health system level diagnosis delay (from contact with first HCP to TB diagnosis) and treatment initiation delays (from diagnosis to treatment initiation). Total delay was the sum of patient level, health system level diagnosis delay and treatment initiation delays.

**Results:**

We included 234 ACF-diagnosed and 231 PCF-diagnosed patients. When compared to PCF, ACF patients were relatively older (≥65 years, 14% versus 8%, p = 0.041), had no formal education (57% versus 36%, p<0.001), had lower monthly income per capita (median 13.1 versus 15.7 USD, p = 0.014), were more likely from rural areas (92% versus 81%, p<0.002) and residing far away from the sputum microscopy centres (more than 15 km, 24% versus 18%, p = 0.126). Fewer patients had history of significant loss of weight (68% versus 78%, p = 0.011) and sputum grade of 3+ (15% versus 21%, p = 0.060). Compared to PCF, HCP visits among ACF patients was significantly lower (median one versus two HCPs, p<0.001). ACF patients had significantly lower health system level diagnosis delay (median five versus 19 days, p = 0.008) and the association remained significant after adjusting for potential confounders. Patient level and total delays were not significantly different.

**Conclusion:**

*Axshya SAMVAD* linked the most impoverished communities to TB care and resulted in reduction of health system level diagnosis delay.

## Introduction

Tuberculosis (TB) is the world’s leading cause of death among infectious diseases. In 2017, there were an estimated 10 million new patients and 1.6 million deaths due to TB [1]. World Health Organization’s ‘End TB strategy’ emphasizes on early diagnosis and treatment which is vital for effective TB management [2,3]. Delays in diagnosis and treatment initiation can result in severe clinical presentation, increased disease transmission and unfavourable outcomes including death [4–9]. Therefore, finding patients early has the potential to reduce TB transmission.

Globally, the epidemiological impact of passive TB case finding (PCF i.e., detecting patients at health facilities among persons who recognize their symptoms and seek medical care on their own) has been inadequate [10–13]. For PCF to be effective, community awareness should be high; health care facilities should be accessible and have appropriate diagnostic tools; and the activity should be complemented with health facility-based systematic screening component or supported by advocacy, communication and social mobilisation (ACSM) and active case finding in marginalised or vulnerable groups (ACF–defined as systematic screening for TB applied outside of health facilities) [13]. In the South African context, mathematical modelling showed that ACF among marginalised or vulnerable groups was likely to have more impact on reducing TB transmission than expanding PCF [14].

India has the highest burden of TB [1,15]. Despite significant gains made by India’s revised National TB control programme (RNTCP) in terms of lives saved, India still accounts for one-third of ‘missing’ 4.3 million patients with TB globally [16,17]. In line with the strategic vision of RNTCP (2012–2017) [18], project *Axshya* (meaning ‘free of TB’) was implemented in India by South-East Asia Office (New Delhi, India) of the International Union against Tuberculosis and Lung Disease (The Union) to enhance the reach and visibility of RNTCP services among vulnerable and marginalized populations and mitigate the impact of TB on the country through ACSM and ACF. Funded by The Global Fund against AIDS, TB and Malaria since 2010, it covered 285 districts spread across 19 states (as in 2017) [19–21].

*Axshya SAMVAD* (sensitization and advocacy in marginalised and vulnerable areas of the district) is the ACF strategy. *SAMVAD* in Sanskrit language means ‘conversation’. In this project, trained community volunteers visited households, educated the members on TB and screened them for TB symptoms. It resulted in detection of a large number of persons with presumptive pulmonary TB and sputum smear positive TB [22]. However, whether *Axshya SAMVAD* identified cases earlier when compared to PCF alone is unknown [23].

Overall individual and community-level benefits from screening for active TB disease remains uncertain [10–12]. One of the four criteria to assess the effectiveness of any screening strategy for active TB is “*does screening for tuberculosis disease identify cases earlier*?*”* [23]. A systematic review (2013) suggested that screening found cases earlier and with less severe disease, but this might be due to more sensitive diagnostic methods used in the studies than routine programmes that implemented PCF [23].

Therefore, this study was conducted among new sputum smear positive TB patients from marginalised and vulnerable populations with the primary objective to determine the effect of *Axshya SAMVAD* on various delays (from eligibility for sputum examination to treatment initiation) when compared to PCF. Secondary objectives were to compare the patient characteristics and health care seeking [24].

## Methods

### Study design

This was an observational analytic study.

### Study setting

#### India’s national TB programme—RNTCP (2016–17)

RNTCP infrastructure included national, state, district and sub-district level administrative units (called as TB units (TUs)—one for 250 000 to 500 000 population) and designated microscopic centers (DMCs–one for 50 000 to 100 000 population) for sputum microscopy [25]. Laboratory registers maintained at the DMCs contained details of each presumptive TB patient who underwent sputum smear microscopy and TB registers maintained at each TU indicated the number of TB patients treated and registered under RNTCP [26].

#### *Axshya SAMVAD* under project *Axshya* (2016–17)

In consultation with the State TB programme, *Axshya* districts and *Axshya* TUs were identified. Even within an *Axshya* TU, activities (including *Axshya SAMVAD*) were preferentially targeted towards marginalised and vulnerable populations (**S1 Annex)**. Each *Axshya* district had a district coordinator who was supervised by the assistant project manager, the state technical consultant and project management unit (PMU) at New Delhi, India.

Technical and operational guidelines for *Axshya SAMVAD* (2016–17) are provided in **S1 Annex**. It was conducted with the support of trained community volunteers (*Axshya mitras*, meaning friends of *Axshya* in Hindi) from local grass root level non-governmental organizations in coordination with district and TU level RNTCP staff. The district coordinator provided one-day training to *Axshya mitras* in identifying TB symptoms using the symptomatic verbal screening criteria (more than 2 weeks of cough, evening rise in temperature, loss of appetite, and loss of weight (any one)) and on collection of quality sputum samples. During house-to-house visits, presumptive TB patients were identified and referred to the nearest DMCs for sputum examination. In case the referral failed, *Axshya mitras* provided sputum collection and transport (SCT) services for patients [27]. Activity-based honorarium was provided to *Axshya mitras* for every house visit made and every SCT done with in-built quality control mechanisms (sputum positivity rate of 7% for SCT) [19].

### Study population and sampling

**Fig 1:**
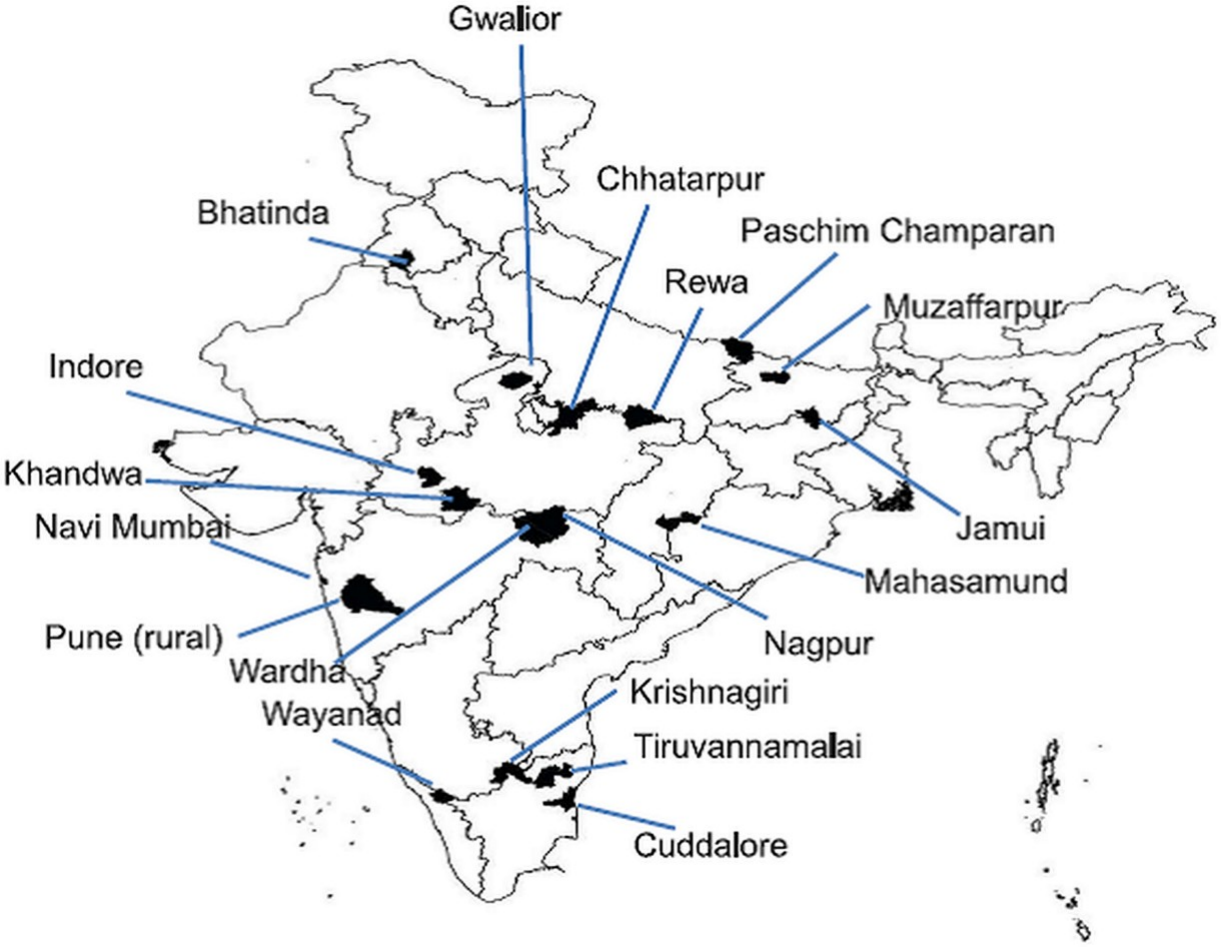
Map of India depicting the randomly sampled *Axshya* districts (n = 18) under *Axshya SAMVAD* study, India (2016–17) [24]*. *SAMVAD–*sensitization and advocacy in marginalised and vulnerable areas of the district. *Axshya SAMVAD*–an active case finding strategy under project *Axshya* implemented by The Union, South East Asia office, New Delhi, India, across 285 districts of India. * Reprinted from Shewade HD et al [24] under a CC BY license, with permission from International Union Against Tuberculosis and Lung Disease (The Union), Copyright The Union 2017.

All sputum smear positive TB patients newly registered for treatment between March 2016 and February 2017 and belonging to marginalised and vulnerable populations in *Axshya* districts were the study population. Eighteen study districts (from seven states) were selected among the *Axshya* districts of India using simple random sampling **(Fig 1)**. The sampling frame for these districts excluded districts from north-eastern India (due to logistic issues in conducting data collection in hilly terrain).

At the beginning of every month (starting April 2016 up to March 2017) in every study district, the district coordinator prepared a list of new sputum smear positive TB patients (registered in previous month). The patient list (in the form of unique identifier—state code-district code-TU code-year-registration number) was updated in an Excel-based (Redmond, WA, USA) study participant enrolment tool shared using cloud-based open-access technologies and classified into three groups: exposed; unexposed and eligible; and unexposed but ineligible [24].

Operational definition for each group is summarized in **Table 1.** To summarize, ‘exposed’ group included patients identified through ACF (*Axshya SAMVAD* / ACF group) and ‘unexposed’ group included patients that were identified by routine case finding mechanisms within the programme (non-*Axshya SAMVAD* / PCF group). ‘Unexposed and ineligible’ group contained patients with mixed/contaminated exposure to *Axshya SAMVAD*. In other words these patients were identified through PCF but *Axshya* SAMVAD activity had been conducted in the village before date of diagnosis [24].

**Table 1:** Operational definition of study participants and sampling methodology in *Axshya SAMVAD* study, India (2016–17) [24]*.

Terminology	Definition
**Study participant**	New smear positive TB patients registered for treatment and belonging to marginalised population in the district
**Study participant–Exposed**	New smear positive TB patients diagnosed through *Axshya SAMVAD* i.e., participants’ residence belongs to a village / urban ward where *Axshya SAMVAD* was conducted before the date of diagnosis and there is clear documentation in the project records that the patient was identified by *Axshya SAMVAD*.
**Study participant–Unexposed and eligible**	New smear positive TB patients (detected through passive case finding) and belong to a village / urban ward where *Axshya SAMVAD* was not conducted (ever) before the date of diagnosis.
**Study participant–Unexposed and ineligible**	New smear positive TB patients (detected through passive case finding) but belonged to a village where *Axshya SAMVAD* was conducted (ever) before date of diagnosis. In such patients, it was challenging to rule out exposure to *Axshya SAMVAD* and hence was excluded from the study.
**Sampling**	All the ‘exposed’ were enrolled into the study, an equal number from the list ‘unexposed and eligible’ were randomly enrolled as ‘unexposed’ (1:1 ratio, exposed: unexposed) and all the ‘unexposed but ineligible’ were excluded from the study.

*SAMVAD–*sensitization and advocacy in marginalised and vulnerable areas of the district

*Axshya SAMVAD–*an active case finding strategy under project *Axshya* implemented by The Union, South East Asia office, New Delhi, India, across 285 districts of India

* Reprinted with modification from Shewade HD et al [24] under a CC BY license, with permission from International Union Against Tuberculosis and Lung Disease (The Union), Copyright The Union 2017

The principal investigator (using the study participant enrolment tool) enrolled all the ‘exposed’ patients into the study. An equal number from the list ‘unexposed and eligible’ were enrolled as ‘unexposed’ (1:1 ratio, exposed: unexposed) using simple random sampling. All the ‘unexposed but ineligible’ were excluded from the study. The details of this sampling have been provided elsewhere [24].

### Operational definition of delay

**Fig 2:**
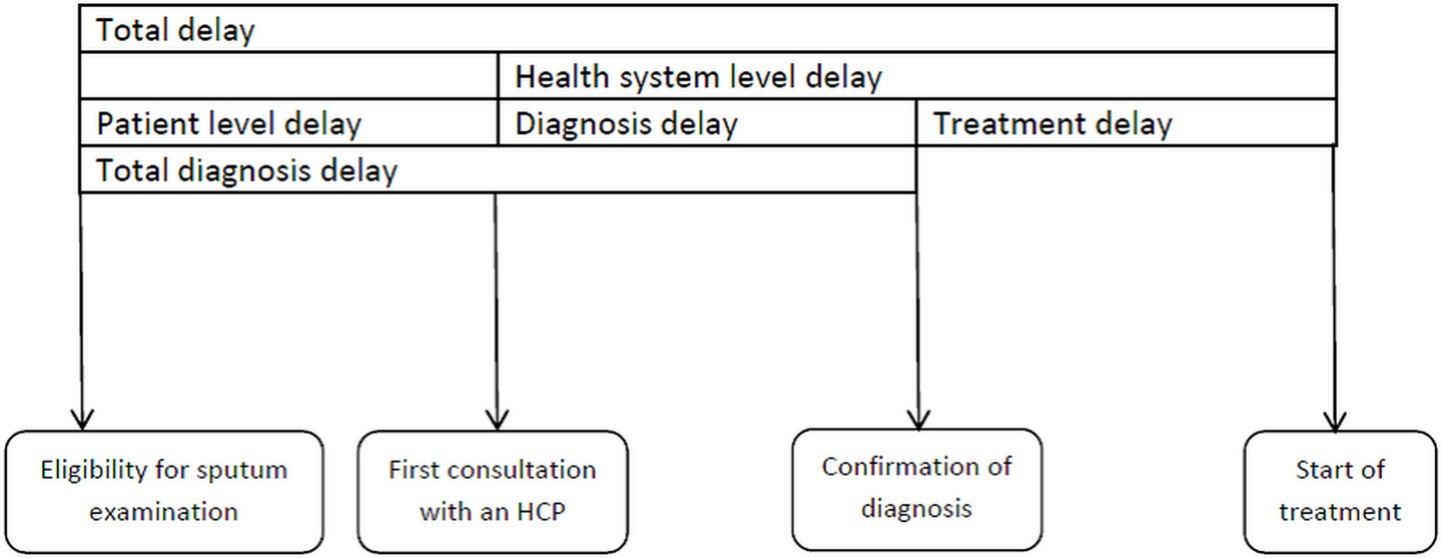
Conceptual framework on definitions of delay before treatment initiation among newly registered sputum postive TB patients, *Axshya SAMVAD* study, India (2016–17) * [28]. * Reprinted with modification from Sreeramareddy CT et al [28] under a CC BY license, with permission from International Union Against Tuberculosis and Lung Disease (The Union), Copyright The Union 2014. *SAMVAD–*sensitization and advocacy in marginalised and vulnerable areas of the district. *Axshya SAMVAD*–an active case finding strategy under project *Axshya* implemented by The Union, South East Asia office, New Delhi, India, across 285 districts of India. HCP–Health Care Providers.

Total delay (in days) was defined as the period from eligibility for sputum examination to treatment initiation. ‘Eligibility for sputum examination’ was defined as ‘fifteenth day of continuous cough/fever or the day of the first episode of haemoptysis (whichever was earlier)’. Total delay was divided into patient level delay (from eligibility for sputum examination to first visit to a health care provider (HCP)) and health system level delay (from first visit to an HCP to date of treatment initiation). Health system level delay was further classified into diagnosis and treatment initiation delay based on the date of diagnosis (sputum examination at DMC). Total diagnosis delay was defined as the sum of patient level delay and health system level diagnosis delay (from eligibility for sputum examination to diagnosis) **(Fig 2).** HCP included qualified modern medicine/allopathic doctors (public or private), qualified alternate medicine doctors (public or private), qualified paramedical workers and unqualified health care providers.

### Data collection

#### Questionnaire

The questionnaire was divided into two parts. Part I contained variables that were extracted from reviewing TB treatment register, treatment card and project *Axshya* records (**S2 Annex**). Part II was an interviewer administered structured closed-ended questionnaire (**S3 Annex**).

Key variables in part I included: exposure status (*Axshya SAMVAD* or non-*Axshya SAMVAD*), age, gender, residence (urban/rural), distance of residence (in km) from nearest DMC, sputum result at diagnosis, weight, HIV status, diabetes mellitus and dates of diagnosis and treatment initiation.

Key variables in part II included some information on self-reported patient’s status at diagnosis [education, occupation, monthly household income per capita, alcohol intake, smoking status (consumption of alcohol / smoke form of tobacco at least once during 30 days before diagnosis was considered as ‘yes’) and TB or TB death in household (ever)] and patient’s health seeking between eligibility for sputum examination and diagnosis [date of eligibility for sputum microscopy, date and type of first HCP visited, total number of HCPs visited, history of fever / haemoptysis / significant weight loss and whose advice led to eventual sputum examination (at DMC)].

The eventual visit to DMC for diagnosis was also considered as a visit to HCP for diagnosis. In *Axshya SAMVAD* group, among patients undergoing SCT, if there wasn’t any visit to an HCP before diagnosis, then number of HCPs visited was recorded as zero. Among referred patients, if there wasn’t any visit to an HCP, the number of visits was recorded as ‘one’ (the visit to DMC for diagnosis).

#### Procedure

Data were collected between April 2016 and June 2017. District coordinators were expected to complete data collection for Part I within one month of enrolment and district-level supervisors (either assistant project managers or state technical consultants) to complete Part II within two months of enrolment.

Part II of the questionnaire was available and administered by interviewer in local language as understood by the patient. Interviews were conducted at the patient’s residence after fixing an appointment over phone or through a village level health worker in the village. All interviews were audio recorded. Scanned forms of part I and II and audio files were shared with the principal investigator (HDS) using the cloud-based open-access technologies [24]. The interviewers were project staff who piggy-backed on their routine supervisory visits to complete the interviews. PMU level supervisors pitched in to complete the interviews if the district level supervisors were not able to incorporate it in their routine schedules.

#### Monitoring of data collection and quality control

Data collectors were trained in February 2016. TeamViewer software, being a remote-control application, was helpful for trouble shooting, training new staff or retraining the existing staff. [24]

Indicators pertaining to timeliness and quality of data collection for Part I and Part II were followed up by district level and PMU level supervisors respectively in an Excel-based (Redmond, WA, USA) patient-wise monitoring tool shared using the cloud-based open-access technologies [24]. Ten percent of audio records were randomly marked for quality check and assessed by PMU level supervisors. Repeat record review and/or interviews were conducted if the data quality was suboptimal. If any project staff identified that an enrolled study participant had to be excluded from the study (mostly due to initial misclassification), it was flagged in the Excel-based (Redmond, WA, USA) patient-wise monitoring tool for review by the principal investigator (HDS) [24].

### Analysis and statistics

#### Sample size

We assumed that there will be at least a difference of five days in the total delay between patients detected by *Axshya SAMVAD* and PCF. Therefore, we needed a sample size of 284 each in *Axshya SAMVAD* and non-*Axshya SAMVAD* groups (1:1 ratio) assuming a SD of 15 in each group, 5% alpha error, 80% power and design effect of two (cluster selection of districts). Anticipating a non-response of 15%, we aimed to enrol 325 in study participants in each group (nMaster sample size calculator version 1.0 software, Christian Medical College, Vellore, India).

#### Data management and analysis

Data were double entered and validated using EpiData entry software (version 3.1, EpiData Association, Odense Denmark) [24]. Descriptive and unadjusted inferential analysis was done using EpiData analysis software (version 2.2.2.183 EpiData Association, Odense Denmark) and adjusted analysis was done using STATA (version 12.1, copyright 1985–2011 StataCorp LP USA).

Patient characteristics, number of HCPs visited, type of first HCP visited (if any), whose advice eventually led to sputum examination and various types of delays were summarized using frequency/proportion, mean (SD) or median (IQR) and compared across *Axshya SAMVAD* and non-*Axshya SAMVAD* groups. Chi square test was used for comparison if the variables were categorical. Unpaired t test and Mann Whiney U test were used for continuous variables if their distribution were normal and non-normal, respectively.

We also did a confounder adjusted analysis for the association between *Axshya SAMVAD* and various types of delays using linear regression after adjusting for clustering at district level. Six models were built: one for each type of delay. Delay variable in each model was log transformed (outcome of interest) as it was not normally distributed. *Axshya SAMVAD* status (yes) was the exposure of interest (reference was ‘no’). Variables were considered in the linear regression model if they were associated with the outcome variable (p<0.20) **(S1 Table).** Association was summarized (inferred) using Beta coefficient (0.95 CI). The Beta coefficient indicated the adjusted mean difference of outcome between the category of interest and the reference category.

There are concerns in applying and interpreting the results of hypothesis testing in a log-transformed data on actual data (non-log-transformed) [29]. Hence, we also determined a confounder-adjusted association between *Axshya SAMVAD* and delay variable using generalised linear models (Poisson regression, outcome of interest was delay more than or equal to median). Potential confounders were restricted to variables that were associated with both the outcome (p<0.2) and the exposure (p<0.05 or programmatically/clinically significant difference) **(S2 Table)**. In each delay model (n = 6), the association was summarized (inferred) using adjusted prevalence ratios (0.95 CI). P value less than 5% was considered as statistically significant.

Irrespective of the type of model (linear regression or generalised linear model), age and gender were considered as potential confounders irrespective of their unadjusted p values. Sputum smear status and history of weight loss, fever or haemoptysis were excluded as we do not expect these to confound the association between *Axshya SAMVAD* exposure and delay. Variables in the causal pathway between *Axshya SAMVAD* exposure and delay (number of HCPs visited and type of first HCP visited) were also excluded. Diabetes (large data missing) and HIV status (only one was positive) were also not considered for the adjusted analysis **(S3 Table).**

### Ethics

Ethics approval was obtained from the Ethics Advisory Group of The Union, Paris, France (EAG number 15/15, dated 28 September 2015). The study was conducted after receiving approvals from the State TB Officers of Tamil Nadu, Kerala, Maharashtra, Madhya Pradesh, Chattisgarh, Bihar and Punjab. Written informed consent was taken from the study participants (from parents/guardians if less than 18 years) and the consent process was approved by the ethics committee.

## Results

### Study participant enrolment

**Fig 3:**
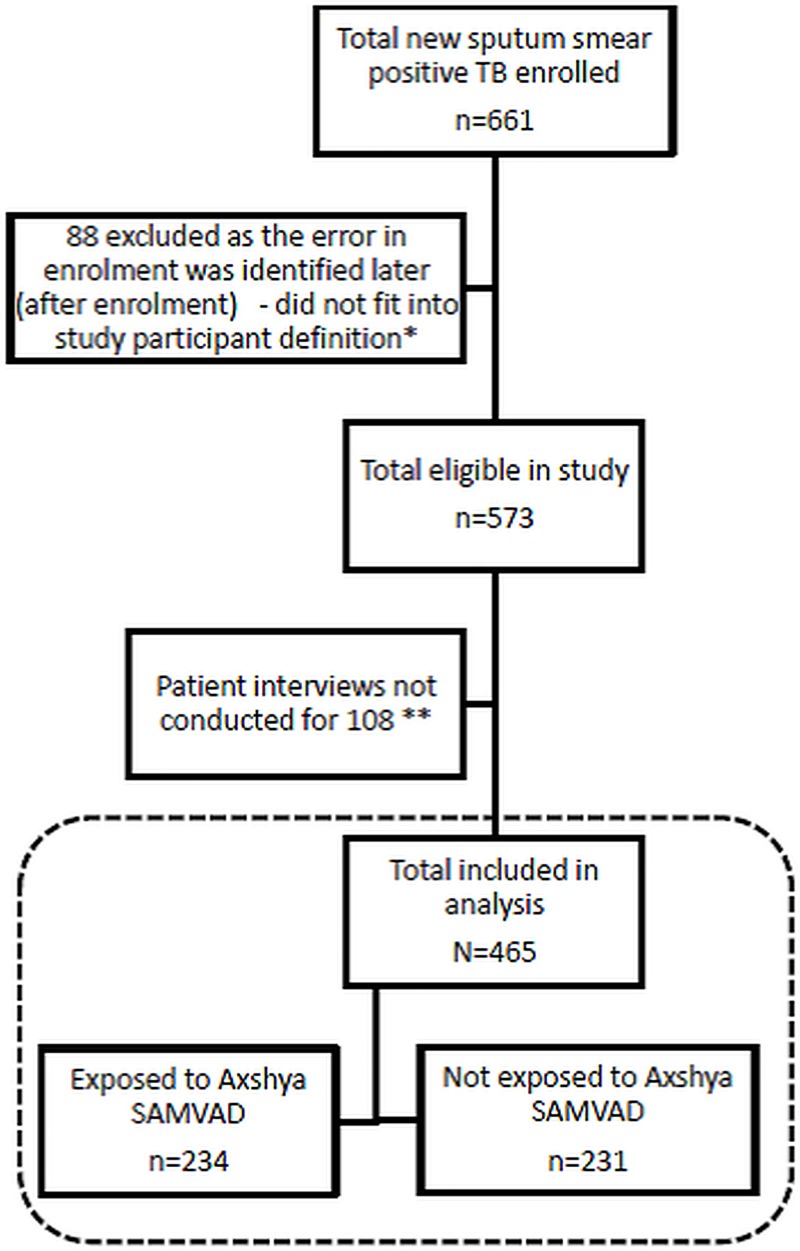
Flow chart depicting study participant enrolment in *Axshya SAMVAD* study across 18 randomly sampled districts in India (2016–2017). *SAMVAD–*sensitization and advocacy in marginalised and vulnerable areas of the district. *Axshya SAMVAD*–an active case finding strategy under project *Axshya* implemented by The Union, South East Asia office, New Delhi, India, across 285 districts of India. *32 were due to errors during record review (either patient turned out to be previously treated or sputum smear negative TB; 56 patients were recorded as new patients but turned out to be previously treated during patient interviews. ** due to patient non-availability during visit to residence.

Study participant enrolment has been depicted in **Fig 3**. Of 661 enrolled, 88 were excluded later as they did not fit into study participant definition. Of 573 eligible, patient interviews were not conducted for 108 (due to patient non-availability during visit to residence). When compared to those interviewed (n = 465), those not interviewed had significantly lower proportion of patients registered through *Axshya SAMVAD*, were more likely from rural areas and with sputum grading of 3+ at diagnosis **(S4 Table)**.

A total of 465 were included in the final analysis: 234 belonged to *Axshya SAMVAD* group and 231 to non-*Axshya SAMVAD* group **(Table 2 and Fig 3)**. Time taken between enrolment and interview has been summarized in **S5 Table.** Among 234 in *Axshya SAMVAD* group, 217 (79%) eventually underwent SCT.

**Table 2:** Distribution of study participants across the 18 randomly sampled districts in India, *Axshya SAMVAD* study, 2016–17 [n = 465].

States	Districts	Total	*Axshya SAMVAD* group	Non-*Axshya SAMVAD* group
**Total**		**465**	**234**	**231**
Punjab	Bhatinda	5	2	3
Bihar	Paschima Champaran	101	50	51
	Muzaffarpur	28	15	13
	Jamui	32	17	15
Madhya Pradesh	Chhatarpur	10	8	2
	Rewa	76	39	37
	Gwalior	54	28	26
	Indore	7	4	3
	Khandwa	13	7	6
Tamil Nadu	Krishnagiri	7	2	5
	Cuddalore	44	20	24
	Tiruvannamalai	9	4	5
Chattisgarh	Mahasamund	24	11	13
Maharashtra	Wardha	10	2	8
	Nagpur Municipal	7	5	2
	Navi Mumbai	9	4	5
	Pune Rural	22	12	10
Kerala	Wayanad	7	4	3

*SAMVAD–*sensitization and advocacy in marginalised and vulnerable areas of the district

*Axshya SAMVAD–*an active case finding strategy under project *Axshya* implemented by The Union, South East Asia office, New Delhi, India, across 285 districts of India

### Comparison of patient characteristics

Socio-demographic, clinical and health system characteristics in *Axshya SAMVAD* and non-*Axshya SAMVAD* group are depicted in **Table 3.** When compared to non-*Axshya SAMVAD*, patients in *Axshya SAMVAD* group had higher age (mean 40 versus 44 years, p = 0.003), higher proportion with no formal education (36% versus 57%, p<0.001), lower monthly household per capita income (median 15.7 versus 13.1 USD, p = 0.014), lower proportion with loss of weight (78% versus 68%, p = 0.032), lower proportion with sputum grade 3+ (21% versus 15%, p = 0.068) and higher proportion with residence in rural area (81% versus 92%, p<0.001) and residing more than 15 km from the DMC (18% versus 24%, p = 0063).

**Table 3:** Baseline characteristics of patients with new sputum smear positive TB enrolled in *Axshya SAMVAD* study across 18 randomly sampled districts in India, 2016–17 (n = 465).

Variable		Total [N = 465]	*Axshya SAMVAD* group	Non-*Axshya SAMVAD* group	p*
			[N = 234]	[N = 231]	
		n (%)	n (%)	n (%)	
**Socio-demographic characteristics**					
Age categories (years)					
	15–44	251 (54)	111 (47)	140 (61)	0.009
	45–64	163 (35)	91 (39)	72 (31)	
	≥65	50 (11)	32 (14)	18 (8)	
	Missing	1 (<1)	0 (0)	1 (<1)	
	Mean (SD)	42 (17)	44 (17)	40 (17)	0.003
Gender					
	Male	307 (66)	153 (65)	154 (67)	0.721
	Female	157 (34)	81 (35)	76 (33)	
	Missing	1 (<1)	0 (0)	1 (<1)	-
Residence					
	Urban	58 (12)	17 (7)	41 (18)	<0.001
	Rural	402 (87)	214 (92)	188 (81)	
	Missing	5 (1)	3 (1)	2 (1)	
Education					
	No formal education	217 (47)	133 (57)	84 (36)	<0.001
	Less than primary	67 (14)	30 (13)	37 (16)	
	Up to secondary	149 (32)	57 (24)	92 (40)	
	Higher secondary and above	30 (7)	13 (6)	17 (7)	
	Missing	2 (<1)	1 (<1)	1 (<1)	
Occupation					
	Unemployed	59 (13)	31 (13)	28 (12)	0.283
	Studying	24 (5)	8 (3)	16 (7)	
	Homemaker	82 (18)	45 (19)	37 (16)	
	Daily wage labour	178 (38)	95 (41)	83 (36)	
	Employed-not daily wage	113 (24)	52 (22)	61 (26)	
	Missing	9 (2)	3 (1)	6 (3)	
Monthly income					
per capita (USD)**	Median (IQR)	15.7 (7.4, 31.4)	13.1 (6.4, 23.6)	15.7 (7.9, 31.4)	0.014
**Clinical characteristics**					
TB in household					
in the past	Yes	116 (25)	54 (23)	62 (27)	0.321
	No	347 (75)	180 (77)	167 (72)	
	Missing	2 (<1)	0 (0)	2 (1)	
TB death in					
household	Yes	51 (11)	27 (11)	24 (10)	0.704
	No	413 (89)	207 (89)	206 (89)	
	Missing	1 (<1)	0 (0)	1 (<1)	
History of					
fever***	Yes	350 (75)	170 (73)	180 (78)	0.231
	No	105 (22)	58 (25)	47 (20)	
	Missing	10 (3)	6 (2)	4 (2)	
History of weight					
loss***	Yes	340 (73)	159 (68)	181 (78)	0.032
	No	113 (24)	66 (28)	47 (20)	
	Missing	12 (3)	9 (4)	3 (2)	
History of					
haemoptysis***	Yes	119 (26)	60 (25)	59 (26)	0.937
	No	336 (72)	168 (72)	168 (73)	
	Missing	10 (2)	6 (3)	4 (1)	
Current Smoker^					
	Yes	113 (24)	65 (28)	48 (21)	0.122
	No	343 (74)	164 (70)	179 (77)	
	Missing	9 (2)	5 (2)	4(2)	
Current alcohol intake^					
	Yes	130 (28)	61 (26)	69 (30)	0.419
	No	327 (70)	168 (72)	159 (69)	
	Missing	8 (2)	5 (2)	3 (1)	
Sputum grading					
	3+	83 (18)	34 (15)	49 (21)	0.068
	Scanty/1+/2+	365 (79)	190 (81)	175 (76)	
	Positive not quantified	17 (4)	10 (4)	7 (3)	
Weight in kg					
	<30	8 (2)	6 (2)	3 (1)	0.540
	30–44.9	200 (43)	102 (44)	98 (42)	
	≥45	96 (21)	44 (19)	52 (23)	
	Missing	161 (35)	83 (35)	78 (34)	
	Mean (SD)	41 (7)	41 (6)	41 (7)	0.781
HIV status^^					
	Positive	1 (<1)	0 (0)	1 (<1)	-
	Negative	287 (59)	143 (61)	144 (62)	
	Missing	177 (38)	91 (39)	86 (37)	
DM status					
	DM	9 (2)	4 (2)	5 (2)	0.784
	Not DM	171 (37)	84 (36)	87 (38)	
	Missing	285 (61)	146 (62)	139 (60)	
**Health system characteristics**					
Distance of					
residence from	≤5	118 (25)	50 (21)	68 (29)	0.063
DMC in km	6–10	144 (31)	80 (34)	64 (28)	
	11–15	107 (23)	49 (21)	58 (25)	
	>15	96 (21)	55 (24)	41 (18)	
	Median (IQR)	10 (5,15)	10 (6, 15)	10 (5, 14)	0.090

Column percentage

TB–tuberculosis; *SAMVAD*–sensitization and advocacy in marginalised and vulnerable areas of the district; SD–standard deviation; USD–US dollar; HIV–human immunodeficiency virus; DM–diabetes mellitus; DMC–designated microscopy centre; IQR–interquartile range

*Axshya SAMVAD*–an active case finding strategy under project *Axshya* implemented by The Union, South East Asia office, New Delhi, India, across 285 districts of India

*p value calculated after excluding missing values, chi square test / independent t test / mann whitney U test

**Pre-TB income, average Indian rupee to USD conversion rate in Jan 2018 (1USD = 63.6 Indian rupees), Indian rupee value used for calculating p value

*** history of fever/significant weight loss/haemoptysis between eligibility for sputum examination and diagnosis

^ consumption of alcohol/smoke form of tobacco anytime in the month before date of diagnosis

^^number with HIV very low (n = 1); hence, p value not calculated

### Health care seeking

Health care seeking in *Axshya SAMVAD* and non-*Axshya SAMVAD* group is depicted in **Table 4**. In *Axshya SAMVAD* group, 52 (22%) did not visit an HCP, 60 (25%) first visited a public facility doctor and 113 (48%) first visited a private facility (qualified doctor or unqualified HCP). When compared to non-*Axshya SAMVAD* group, patients in *Axshya SAMVAD* group had lower number of median visits to an HCP (two versus one); higher proportion with zero visits (0% versus 22%) and lower proportion with three or more visits (39% versus 16%); and lower proportion with first visit to private HCP (57% versus 48%).

*Axshya SAMVAD* activity was done before first visit to an HCP in 44% (104/234) patients. Among these 104 patients, 89 underwent SCT and 15 were referred. Despite this, zero number of HCP visits was seen only in 52 patients. This was due to two reasons. First, there was a median (IQR) delay of five (0, 9) days between date of *Axshya SAMVAD* activity and date of SCT/referral and a median (IQR) delay of nine (1, 36) days between referral and diagnosis during which some patients might have visited HCPs. Second, of 234 patients in *Axshya SAMVAD* group, in 188 (80%) the advice by *Axshya mitra* eventually led to sputum examination. In the remaining instances, patients said that they consulted HCPs (after identification by *Axshya mitra*) before getting diagnosed **(Table 5)**.

**Table 4:** Visits to health care provider^#^ from eligibility for sputum examination^ to diagnosis among patients with new sputum smear positive TB enrolled in *Axshya SAMVAD* study across 18 randomly sampled districts in India, 2016–17 (n = 465).

Variable			Total	*Axshya SAMVAD* group	Non-*Axshya SAMVAD* group	P value*
			[N = 465]	[N = 234]	[N = 231]	
			n (%)	n (%)	n (%)	
Number of visits						
	Zero		52 (11)	52 (22)	0 (0)	<0.001
	One		137 (30)	67 (29)	70 (30)	
	Two		134 (29)	64 (28)	70 (30)	
	Three or more		128 (27)	39 (16)	89 (39)	
	Missing		14 (3)	12 (5)	2 (1)	
	Median (IQR)		2 (1,3)	1 (1,2)	2 (1,3)	<0.001
Health care provider						
first visited	None visited		52 (11)	52 (22)	0 (0)	<0.001
	Unqualified private		108 (23)	56 (24)	52 (23)	
	Qualified					
		Public facility doctor	158 (34)	60 (26)	98 (42)	
		Private doctor	135 (29)	57 (24)	78 (34)	
		Others**	6 (1)	3 (1)	3 (1)	
	Missing		6 (1)	6 (2)	0 (0)	

Column percentage; total may not be 100% as these have been rounded of nearest whole number

TB–tuberculosis; *SAMVAD*–sensitization and advocacy in marginalised and vulnerable areas of the district; IQR–interquartile range

*Axshya SAMVAD*–an active case finding strategy under project *Axshya* implemented by The Union, South East Asia office, New Delhi, India, across 285 districts of India

^**#**^ Health care provider included qualified modern medicine/allopathic doctors (public or private), qualified alternate medicine doctors (public or private), qualified paramedical workers and unqualified health care providers.

^fifteenth day of cough/fever or day of haemoptysis whichever is earlier

*p value calculated after excluding missing values, chi square / mann whitney U test

**others could be could be village level health staff or pharmacist of a chemist shop or facility level paramedic

**Table 5:** ‘Whose advice eventually led to sputum examination’: response of patients with new sputum smear positive TB enrolled in *Axshya SAMVAD* study across 18 randomly sampled districts in India, 2016–17 (n = 465).

	Total	*Axshya SAMVAD* group	Non-*Axshya SAMVAD* group
	[N = 465]	[N = 234]	[N = 231]
	n (%)	n (%)	n (%)
Axshya ‘Mitra’*	200 (43.0)	188 (80.3)^	12 (5.2)^
Other / somebody else	73 (15.7)	14 (6.0)	59 (25.5)
Private qualified doctor	53 (11.4)	13 (5.6)	40 (17.3)
Government doctor	58 (12.5)	7 (3.0)	51 (22.1)
Family member	26 (5.6)	5 (2.1)	21 (9.1)
Missing	10 (2.2)	4 (1.7)	6 (2.6)
Rural health care provider**	13 (2.8)	2 (0.9)	11 (4.8)
Community member	18 (3.9)	1 (0.4)	17 (7.4)
Self-nobody advised	4 (0.9)	0 (0.0)	4 (1.7)
Paramedical staff in community***	10 (2.2)	0 (0.0)	10 (4.3)

Column percentage

TB–tuberculosis; *SAMVAD*–sensitization and advocacy in marginalised and vulnerable areas of the district (active case finding strategy)

*community volunteer who implements *Axshya SAMVAD*

^despite identification as presumptive pulmonary TB by *Axshya Mitra*, 20% TB patients in “Axshya *SAMVAD*” group still considered taking suggestion from others before getting the sputum examination done, *Axshya mitras* also contributed at some point in diagnosis of five percent cases in “Non-*Axshya SAMVAD*” group, however this contribution was not during their active case finding activity in the field.

**unqualified health care provider

***included accredited social health activists, auxiliary nurse midwife, *anganwadi* workers

### Comparison of delay

Various types of delays in *Axshya SAMVAD* and non-*Axshya SAMVAD* group are depicted in **Table 6.** When compared to non-*Axshya SAMVAD* group, patients in *Axshya SAMVAD* group had a lower total delay (medina 62 versus 52 days), health system level delay (median 23 versus 16 days) and total diagnosis delay (median 61 versus 45 days); and higher patient level delay (median 10 versus 12 days). These differences were not statistically significant (p = 0.37, p = 0.148, p = 0.131 and p = 0.999, respectively). However, median health system level diagnosis delay and treatment initiation delay were significantly lower and significantly higher by 14 (p = 0.008) and two days (<0.001) respectively in *Axshya SAMVAD* group. Within *Axshya SAMVAD* group, the treatment initiation delay among those referred and underwent SCT was two and four days, respectively: the former was similar to non-*Axshya SAMVAD* group.

**Table 6:** Patient level and health system level delays (in days) from eligibility for sputum examination to treatment initiation among patients with new sputum smear positive TB enrolled in *Axshya SAMVAD* study across 18 randomly sampled districts in India, 2016–17 (n = 465).

Delay type*	Total		*Axshya SAMVAD* group		Non-*Axshya SAMVAD* group		p***
	Assessed (n)**	Median (IQR)	Assessed (n)	Median (IQR)	Assessed(n)	Median (IQR)	
Patient level (a)	455	11 (3,34)	225	12 (3,31)	230	10 (3,43)	0.999
Health system level–diagnosis delay (b)	458	14 (0,71)	229	5 (0,61)	229	19 (1,76)	0.008
Treatment initiation delay (c)	460	3 (1,7)	234	4 (2,8)	231	2 (1,5)	<0.001
Total diagnosis delay (a+b)	459	50 (18, 111)	229	45 (18, 106)	230	61 (20,121)	0.131
Health system level (b+c)	455	25 (6,81)	227	16 (3, 71)	228	23 (5,82)	0.148
Total delay (a+b+c)	456	57 (22,116)	227	52 (22,112)	229	62 (23, 128)	0.370

TB–tuberculosis; *SAMVAD*–sensitization and advocacy in marginalised and vulnerable areas of the district; *Axshya SAMVAD*–an active case finding strategy under project *Axshya* implemented by The Union, South East Asia office, New Delhi, India, across 285 districts of India

*patient level delay from date of eligibility for sputum examination to first health care provider visited; health system level diagnosis delay from date of first health care provider visited to diagnosis; treatment initiation delay from date of diagnosis to treatment initiation

**dates missing for some patients

***Mann Whitney U test

The direction and significance of association between *Axshya SAMVAD* and health system level diagnosis delay did not change after a confounder adjusted analysis in linear regression models. However, the increase in treatment initiation delay in *Axshya SAMVAD* was not found to be statistically significant in adjusted analysis **(Table 7)**. In the corresponding adjusted associations in generalised linear model **(S6 Table)**, total diagnosis delay had a significant association with *Axshya SAMVAD* (23% lower chance of total diagnosis delay ≥50 days when compared to PCF, p = 0.009) while health system level diagnosis delay (18% lower chance of health system level diagnosis delay ≥14 days when compared to PCF, p = 0.050) and treatment initiation delay were not associated (24% higher chance of treatment initiation delay ≥3 days when compared to PCF, p = 0.051).

**Table 7:** Confounder adjusted association between *Axshya SAMVAD* and various types of delays in days (outcome—log transformed) using linear regression after accounting for clustering in districts, *Axshya SAMVAD* study, India, 2016–17 (n = 465)*.

Outcome in the model—type of delay**	Beta coefficient	(0.95 CI)	P value
Patient level delay (a)	-0.08	(-0.46, 0.31)	0.673
(n = 454)			
Health system level–diagnosis delay (b)	-0.48	(-0.93, -0.02)	0.041^
(n = 457)			
Treatment initiation delay (c)	0.25	(-0.07, 0.57)	0.116
(n = 445)			
Total diagnosis delay (a+b)	-0.31	(-0.62, 0.00)	0.052
(n = 458)			
Health system level delay (b+c)	-0.30	(-0.75, 0.14)	0.171
(n = 454)			
Total delay (a+b+c)	-0.20	(-0.50, 0.10)	0.181
(n = 455)			

*SAMVAD–*sensitization and advocacy in marginalised and vulnerable areas of the district

*Axshya SAMVAD–*an active case finding strategy under project *Axshya* implemented by The Union, South East Asia office, New Delhi, India, across 285 districts of India

*Delay variable in each linear regression model was log transformed as it was not normally distributed; for variables that were adjusted for, see S3 Table; complete case analysis was done.

**patient level delay from date of eligibility for sputum examination to first health care provider visited; health system level diagnosis delay from date of first health care provider visited to diagnosis; treatment initiation delay from date of diagnosis to treatment initiation

^statistically significant (p<0.05)

## Discussion

Project *Axshya* was implemented on a very large scale (around half of the districts in India) among marginalised and vulnerable populations in India. *Axshya SAMVAD*, an active case finding strategy under the project, was successful in identifying patients who were comparatively more marginalized and vulnerable and relatively less sick when compared to PCF. *Axshya SAMVAD* reduced delays in diagnosis which was probably mediated through reducing the number of HCPs visited.

### Strengths

This study had many strengths. First, both ACF and PCF patients were tested within the same DMC and same diagnostic algorithm was applied. In addition, the comparison group included patients from the same month and from the marginalized populations of the same area as the *Axshya SAMVAD* patients.

Second, the study participants were representative of their respective reference populations for the following reasons i) all participants registered through *Axshya SAMVAD* were included; ii) randomly sampled patients were included in non-*Axshya SAMVAD* group and the person performing the sampling (principal investigator (HDS)) was blinded to patient characteristics; and iii) patients with potential contamination of exposures were excluded. Third, to reduce interviewer bias (it was not possible to blind the project staff to study participant exposure status), we ensured quality control through audio recording of all the interviews followed by random check by supervisors. Fourth, an innovative resource-efficient model for data collection was used which helped in near real-time data sharing and monitoring in operational settings. Fifth, double data entry and validation minimized data entry errors. Finally, an exhaustive list of potential confounders was available making our adjusted analysis robust.

### Limitations

There were some major limitations. Though target sample size was reached (we enrolled more than 650), because of misclassification (by the project staff or by the programme staff), many patients were excluded **(Fig 3)**. Of those eligible (n = 573), interviews could not be conducted for 108 (19%). These factors restricted our sample size. This along with high dispersion and non-normal distribution of delay variables (more than our assumption for sample size calculations) could be the reason for statistically insignificant results for total delay despite having a difference of more than five days (our assumption). As the distribution of two key variables (from part I of questionnaire: residence (urban/rural) and sputum grade 3+) was not similar in those interviewed and those not interviewed, potential bias cannot be ruled out.

Recall limitation is possible as the interviews were not done immediately after registration. This is of relevance because many variables were self-reported by the study participants. However, there wasn’t any differential recall bias among Axshya and non-Axshya *SAMVAD* groups **(S5 Table).** Overall delay in conducting interviews was due to logistic issues as the research was done in routine settings. As we took registered patients, diagnosed patients that underwent initial loss to follow up were not included in the study. We did not collect information on cough frequency or cough duration. This would have been of added value for demonstrating that clinically stable and less sick patients were diagnosed by *Axshya SAMVAD*.

### Interpretation of key findings

Limitations notwithstanding, this study had some key findings. First, though all the study participants belonged to similar geographic area, the patients detected by *Axshya SAMVAD* were relatively more marginalized and vulnerable when compared to those by PCF. Hence, *Axshya SAMVAD* played a major role in linking the most impoverished for diagnosis and treatment under RNTCP. Clinically, when compared to patients detected by PCF, patients in *Axshya SAMVAD* group were less sick at diagnosis, probably indicating earlier diagnosis. This is in line with the findings of other ACF studies worldwide [30–32].

Second, *Axshya SAMVAD* resulted in reduction of health system level diagnosis delay and total diagnosis delay (**Table 7 and S6 Table**) probably mediated through lower number of health care providers visited. This probably resulted in reduction in total costs incurred by the patient and prevalence of catastrophic costs due to TB diagnosis. However, *Axshya SAMVAD* did not address the issues of intensity and inequity in distribution of catastrophic costs due to TB diagnosis (published elsewhere) [33]. There is scope to further reduce the number of HCPs visited by reducing the delay between the *Axshya SAMVAD* activity and referral / SCT and between referral and diagnosis.

Third, more than half of the patients in *Axshya SAMVAD* group had visited an HCP after being eligible for sputum examination and before *Axshya SAMVAD* exposure. The fact that patient level delay was not different, but health level diagnostic delay was, is also reflective of this missed opportunity for TB diagnosis. In addition, in half of the patients, the first HCP visited was a private HCP (qualified or unqualified): this is also corroborated by a systematic review from India **(S7 Table)** [26].

Finally, the effect of Axshya *SAMVAD* was not seen on reduction of health system level delay. This was probably contributed by the treatment initiation delay among patients undergoing SCT (79% patients in *Axshya SAMVAD* group underwent SCT). The referred patients that reached the DMC were initiated on treatment within 2 days of diagnosis similar to patients detected through PCF. Though SCT has been documented to improve sputum examinations and case detection [34], at patient level, we speculate that patients detected through SCT (after ‘failed referral’) had a lower motivation and seriousness regarding the illness (hence for treatment initiation as well) when compared to those who were referred and reached the DMC. This could be due to non-acceptance of diagnosis, limited awareness regarding TB and acceptability barriers in accessing TB diagnostic services in this group of patients [35–37]. Another reason could be that patients detected by SCT were not available at DMC at the time of diagnosis and hence there might have been delays in communication of sputum microscopy results to the patient. The results had to be collected and shared with the patient by the *Axshya mitra*. Since there weren’t any additional incentives or honoraria for treatment initiation, they might not have prioritised this activity.

While we did not find a significant effect on total delay, a community randomized trial in two rural districts of Ethiopia (2003–04) reported a 35% reduction in total delay beyond 3 months among patients in ACF group when compared to PCF group [38]. On the other hand, another community randomized trial from impoverished settlements in Brazil (2005–06) reported that there was no difference in total delay among patients detected through ACF (door to door campaign) when compared to enhanced case finding (awareness generation and leaflet distribution (enhanced case finding)) [39].

### Implications for policy and practice

#### Implications for project *Axshya*

*Axshya SAMVAD* is doing well in identifying the most marginalised and vulnerable patients and that too early during their course of illness by reducing delays in diagnosis. However, there is some scope for improvement.

The project should consider steps to reduce the delays between i) *Axshya SAMVAD* activity and referral/SCT ii) referral and diagnosis; and iii) diagnosis and treatment initiation. The first two steps have the potential to reduce patient level delays among those who had not visited an HCP at the time of *SAMVAD* activity and further reduce health system level diagnosis delays among those who had visited an HCP at the time of activity. The project is now considering SCT and/or assisted referral (*Axshya mitra* would accompany the patient to the DMC) for all patients without the need for a documented failed referral.

#### Implications for RNTCP

There are two implications for RNTCP. First, the evidence generated that ACF substantially reduces diagnosis delay and prevalence of catastrophic costs due to TB diagnosis in marginalised and vulnerable populations supports the initiative of RNTCP to scale up this activity among marginalised and vulnerable populations in all the districts of India [33,40]. Under project *Axshya*, there were honoraria for *Axshya mitras* and a dedicated project staff at district level with supervisory mechanisms in place. Similarly, appropriate incentives and monitoring and supervisory mechanisms are recommended as RNTCP implements ACF in programme settings.

Second, RNTCP should address the missed opportunity for TB diagnosis during first HCP visit, especially among private HCPs. This has been acknowledged in the national strategic plan (2017–25) and private sector engagement has been identified as one of the four thrust areas [40].

## Conclusion

This study adds to the evidence base favouring active case finding for TB among those with poor access. *Axshya SAMVAD*, an active case finding strategy in community settings among marginalized and vulnerable populations conducted over a large scale in India, provided healthcare equity for vulnerable groups and reduced the diagnosis delay when compared to passive case finding. Project *Axshya* may take steps to further reduce the diagnosis delay through assisted referral and/or SCT without the need for a documented failed referral.

## Supporting information

S1 Table Unadjusted association (p value^) of potential confounders (to be considered in the linear regression models) with *Axshya SAMVAD* exposure (exposure of interest) and various delays* (outcome), *Axshya SAMVAD* study, India, 2016-17(n = 465). (DOCX)

S2 Table Unadjusted association (p value^) of potential confounders (to be considered in generalised linear models) with *Axshya SAMVAD* exposure (exposure of interest) and various delays* (outcome), *Axshya SAMVAD* study, India, 2016-17(n = 465). (DOCX)

S3 Table Variables considered in the models to determine association between delay and *Axshya SAMVAD* exposure, *Axshya SAMVAD* study, India (2016–17)^#^. (DOCX)

S4 Table Comparison of baseline characteristics among study participant whose structured one-to-one interview (part II of questionnaire) was conducted and not conducted, *Axshya SAMVAD* study, India, April 2016 –Mar 2017 (N = 573). (DOCX)

S5 Table Median (IQR) time taken (in days) for completion of data collection for part I (record review) and part II (patient interview at residence) of the questionnaire after study participant enrolment in *Axshya SAMVAD* study across 18 randomly sampled districts in India, April 2016-Mar 2017*. (DOCX)

S6 Table Confounder adjusted association between *Axshya SAMVAD* and various types of delays more than / equal to median (in days) using generalised linear models after accounting for clustering in districts, *Axshya SAMVAD* study, India, 2016-17(n = 465)*. (DOCX)

S7 Table Comparison of various delays [median(IQR)] in days, number of health care providers visited, first health care provider visited among *Axshya SAMVAD* and non-*Axshya SAMVAD* group with a findings from a previous systematic review from India. (DOCX)

S1 Annex Technical and operational guidelines for *Axshya SAMVAD* (2016–17) under project *Axshya*, India. *SAMVAD*–sensitization and advocacy in marginalised and vulnerable areas of the district. *Axshya SAMVAD*–an active case finding strategy under project *Axshya* implemented by The Union, South East Asia office, New Delhi, India, across 285 districts of India.
(PDF)

S2 Annex Part I of the questionnaire used for data collection (record review), *Axshya SAMVAD* study, India (2016–17). *SAMVAD*–sensitization and advocacy in marginalised and vulnerable areas of the district. *Axshya SAMVAD*–an active case finding strategy under project *Axshya* implemented by The Union, South East Asia office, New Delhi, India, across 285 districts of India.
(PDF)

S3 Annex Part II of the questionnaire used for data collection (structured interviewer administered questionnaire), *Axshya SAMVAD* study, India (2016–17). *SAMVAD*–sensitization and advocacy in marginalised and vulnerable areas of the district. *Axshya SAMVAD*–an active case finding strategy under project *Axshya* implemented by The Union, South East Asia office, New Delhi, India, across 285 districts of India.
(PDF)

S4 Annex Dataset including the codebook. (XLSX)
