# Supplementary material for: Patient characteristics, health seeking and delays among new sputum smear positive TB patients identified through active case finding when compared to passive case finding in India
Source: PLoS One. 2019 Mar 13;14(3):e0213345. doi: 10.1371/journal.pone.0213345 (PMC6415860; doi:10.1371/journal.pone.0213345)
Supplement: S1 Table — (DOCX) [file pone.0213345.s001.docx]

**S1 Table. Unadjusted association (p value^) of potential confounders (to be considered in the linear regression models) with *Axshya* *SAMVAD* exposure (exposure of interest) and various delays* (outcome), *Axshya* *SAMVAD* study, India, 2016-17(n=465)**

| **Potential confounder**** | ***Axshya* *SAMVAD* exposure** | **Total delay** | **Diagnosis delay** | **Health system level delay** | **Patient level delay** | **Health system level diagnosis delay** | **Treatment initiation delay** |
| --- | --- | --- | --- | --- | --- | --- | --- |
| Age in years | 0.003 | 0.007 | 0.006 | 0.537 | 0.002 | 0.473 | 0.604 |
| Gender | 0.721 | 0.773 | 0.608 | 0.066 | 0.036 | 0.081 | 0.303 |
| Residence (urban/rural) | <0.001 | 0.685 | 0.728 | 0.457 | 0.245 | 0.132 | 0.070 |
| Education | <0.001 | <0.001 | 0.001 | 0.054 | 0.060 | 0.117 | 0.018 |
| Occupation | 0.283 | 0.002 | 0.004 | 0.002 | 0.608 | 0.006 | 0.049 |
| Monthly income per capita | 0.001 | 0.606 | 0.868 | 0.559 | 0.500 | 0.672 | <0.001 |
| TB in household (ever) | 0.321 | 0.977 | 0.852 | 0.706 | 0.350 | 0.985 | 0.482 |
| TB death in household (ever) | 0.704 | 0.565 | 0.488 | 0.195 | 0.486 | 0.051 | 0.318 |
| Alcohol use | 0.419 | 0.023 | 0.048 | 0.432 | 0.146 | 0.712 | 0.320 |
| Smoking | 0.122 | 0.396 | 0.629 | 0.805 | 0.272 | 0.376 | 0.473 |
| Distance from nearest DMC in km | 0.090 | 0.030 | 0.049 | 0.018 | 0.521 | 0.023 | 0.006 |

TB – tuberculosis; SAMVAD – sensitization and advocacy in marginalised and vulnerable areas of the district (an active case finding strategy)

^^Mann Whitney U test or unpaired t test or Chi square test was used as appropriate

*patient level delay from date of eligibility for sputum examination to first health care provider visited; health system level diagnosis delay from date of first health care provider visited to diagnosis; treatment initiation delay from date of diagnosis to treatment initiation. All delays have been log transformed

**sputum smear status; and history of weight loss, fever or haemoptysis were excluded as we do not expect these to confound the association between Axshya SAMVAD and delay. Rather it is the other way around: delay in diagnosis results in severe sputum grade and clinical features and presentation. Variables in the causal pathway between Axshya SAMVAD and delay (first health care provider type, number of health care providers visited) were also excluded. Diabetes (large data missing) and HIV status (only one was positive) were also not considered for the adjusted analysis.
