# Supplementary material for: Patient characteristics, health seeking and delays among new sputum smear positive TB patients identified through active case finding when compared to passive case finding in India
Source: PLoS One. 2019 Mar 13;14(3):e0213345. doi: 10.1371/journal.pone.0213345 (PMC6415860; doi:10.1371/journal.pone.0213345)
Supplement: S3 Table — (DOCX) [file pone.0213345.s003.docx]

**S3 Table. Variables considered in the models to determine association between delay and *Axshya* *SAMVAD* exposure, *Axshya* *SAMVAD* study, India (2016-17)^#^**

| **Outcome variable in the model** | **Linear regression*** | **Generalised linear model**** |
| --- | --- | --- |
| Health system level diagnosis delay | Age, gender, residence (urban/rural), education, occupation, history of TB related death in household, distance from DMC | Age, gender, residence (urban/rural), education, distance from DMC |
| Treatment initiation delay | Age, gender, residence (urban/rural), education, occupation, monthly income per capita, distance from DMC | Age, gender, residence (urban/rural), education, monthly income per capita, distance from DMC |
| Health system level delay | Age, gender, education, occupation, TB related death in household, distance from DMC | Age, gender, education, distance from DMC |
| Patient level delay | Age, gender, education, alcohol use | Age, gender, distance from DMC |
| Total diagnosis delay | Age, gender, education, occupation, distance from DMC, alcohol use | Age, gender, education, distance from DMC |
| Total delay | Age, gender, education status, occupation, distance from DMC, alcohol use | Age, gender, residence, education, distance form DMC |

DMC – designated microscopy centre

**^#^** In both types of models, complete case analysis was done. Sputum smear status; and history of weight loss, fever or haemoptysis were excluded as we do not expect these to confound the association between Axshya SAMVAD and delay. Rather it is the other way around: delay in diagnosis results in severe sputum grade and clinical features and presentation. Variables in the causal pathway between Axshya SAMVAD and delay (first health care provider type, number of health care providers visited) were also excluded. Diabetes (large data missing) and HIV status (only one was positive) were also not considered for the adjusted analysis.

*Delay variable in each model was log transformed as it was not normally distributed. In addition to Axshya SAMVAD status, age, gender and variables with unadjusted p value <0.2 were considered in the linear regression model.

** Delay variable was categorized based on the median value. In addition to Axshya SAMVAD status, age, gender and variables associated with both the outcome (p<0.2) and exposure (p<0.05 or programmatically / clinically significant difference) were considered in the model.
