# Supplementary material for: Patient characteristics, health seeking and delays among new sputum smear positive TB patients identified through active case finding when compared to passive case finding in India
Source: PLoS One. 2019 Mar 13;14(3):e0213345. doi: 10.1371/journal.pone.0213345 (PMC6415860; doi:10.1371/journal.pone.0213345)
Supplement: S4 Table — (DOCX) [file pone.0213345.s004.docx]

**S4 Table.** **Comparison of baseline characteristics among study participant whose structured one-to-one interview (part II of questionnaire) was conducted and not conducted, *Axshya* *SAMVAD* study, India, April 2016 – Mar 2017 (N=573)**

| **Variable** | **Interview conducted (n=465)** | **Interview not conducted (n=108)** | **P value** |
| --- | --- | --- | --- |
| Exposed to *Axshya* *SAMVAD* [n (%)] | 234 (50) | 42 (39) | 0.03* |
| Rural residence [n (%)] | 402 (87) | 82 (77) | <0.01* |
| Distance of residence from DMC [Median (IQR)] | 10 (5,15) | 10 (4,15) | 0.92^ |
| Age [Mean (SD)] | 42 (17) | 40 (18) | 0.31** |
| Male gender [n (%)] | 307 (66) | 70 (65) | 0.88* |
| Sputum result 3+ at diagnosis | 83 (18) | 9 (9) | 0.02* |
| Weight in kg at diagnosis Mean (SD)] | 41 (7) | 40 (8) | 0.30** |

Column percentage

Only one study participant was HIV positive; DM status missing in programme records for >60% study participant records

*Chi square test; ^Krushkal Wallis test, **Unpaired t test
