# Supplementary material for: Patient characteristics, health seeking and delays among new sputum smear positive TB patients identified through active case finding when compared to passive case finding in India
Source: PLoS One. 2019 Mar 13;14(3):e0213345. doi: 10.1371/journal.pone.0213345 (PMC6415860; doi:10.1371/journal.pone.0213345)
Supplement: S5 Table — (DOCX) [file pone.0213345.s005.docx]

**S5 Table. Median (IQR) time taken (in days) for completion of data collection for part I (record review) and part II (patient interview at residence) of the questionnaire after study participant enrolment in *Axshya* *SAMVAD* study across 18 randomly sampled districts in India, April 2016-Mar 2017***

|  | Total (n=573) | *Axshya* *SAMVAD* (n=276) | Non-*Axshya* *SAMVAD* (n=297) | P value |
| --- | --- | --- | --- | --- |
| Part I | 0 (0,7) | 0 (0,8) | 0 (0,7) | 0.70 |
| Part II | 41 (24,62) | 40 (27, 62) | 41 (24, 62) | 0.92 |

*Part II was not filled for 108 patients (42 – Axshya SAMVAD; 66 – Non-Axshya SAMVAD)

Of 465 patients for whom part II was filled, data collection was done within the target 60 days of enrolment in 332 (71.4%) instances

Part I was filled for all: it was done within the target 30 days in 519 (90.6%) instances
