# Supplementary material for: Patient characteristics, health seeking and delays among new sputum smear positive TB patients identified through active case finding when compared to passive case finding in India
Source: PLoS One. 2019 Mar 13;14(3):e0213345. doi: 10.1371/journal.pone.0213345 (PMC6415860; doi:10.1371/journal.pone.0213345)
Supplement: S6 Table — (DOCX) [file pone.0213345.s006.docx]

**S6 Table. Confounder adjusted association between *Axshya* *SAMVAD* and various types of delays more than / equal to median (in days) using generalised linear models after accounting for clustering in districts, *Axshya* *SAMVAD* study, India, 2016-17(n=465)***

| **Outcome in the model - type of delay**** | **Adjusted prevalence ratio** | **(0.95 CI)** | **P value** |
| --- | --- | --- | --- |
| Patient level delay (a) | 1.08 | (0.89, 1.31) | 0.451 |
| (n=454) |  |  |  |
|  |  |  |  |
| Health system level – diagnosis delay (b) | 0.82 | (0.68, 1.00) | 0.050 |
| (n=457) *** |  |  |  |
| Treatment initiation delay (c) | 1.24 | (0.99, 1.55) | 0.051 |
| (n=445) *** |  |  |  |
| Total diagnosis delay (a+b) | 0.77 | (0.63, 0.94) | 0.009^ |
| (n=458) ** |  |  |  |
| Health system level delay (b+c) | 0.85 | (0.65, 1.09) | 0.201 |
| (n=454) *** |  |  |  |
| Total delay (a+b+c) | 0.84 | (0.69, 1.03) | 0.101 |
| (n=455) *** |  |  |  |

SAMVAD – sensitization and advocacy in marginalised and vulnerable areas of the district

Axshya SAMVAD – an active case finding strategy under project Axshya implemented by The Union, South East Asia office, New Delhi, India, across 285 districts of India

*Delay variable in each generalised linear model (Poisson regression) was categorized based on median value among all study participants; details of confounders included in the adjusted analysis in model for each type of delay are available in S3 Table; complete case analysis was done

**patient level delay from date of eligibility for sputum examination to first health care provider visited; health system level diagnosis delay from date of first health care provider visited to diagnosis; treatment initiation delay from date of diagnosis to treatment initiation

^statistically significant
