# Supplementary material for: Patient characteristics, health seeking and delays among new sputum smear positive TB patients identified through active case finding when compared to passive case finding in India
Source: PLoS One. 2019 Mar 13;14(3):e0213345. doi: 10.1371/journal.pone.0213345 (PMC6415860; doi:10.1371/journal.pone.0213345)
Supplement: S7 Table — (DOCX) [file pone.0213345.s007.docx]

**S7 Table. Comparison of various delays [median(IQR)] in days, number of health care providers visited, first health care provider visited among *Axshya* *SAMVAD* and non-*Axshya* *SAMVAD* group with a findings from a previous systematic review from India**

| **Variable** | ***Axshya* *SAMVAD*** | **Non-*Axshya* *SAMVAD*** | **Systematic review, India (2014)*** |
| --- | --- | --- | --- |
| Health care providers | 1.8 (mean) | 2.7 (mean) | 2.7 (mean) |
| visited | 1 (median) | 2 (median) |  |
| First HCP visited was a private HCP | 48% | 57% | 48% |
| Patient level delay | 12 (3,31) | 10 (3,43) | 18 (14,27) |
| Health system level – diagnosis delay | 5 (0,61) | 19 (1,76) | 31 (25,35) |
| Treatment initiation delay | 4 (2,8) | 2 (1,5) | 3 (2, 4) |
| Total diagnosis delay | 45 (18, 106) | 61 (20,121) | - |
| Health system level | 16 (3, 71) | 23 (5,82) | - |
|  |  |  |  |
| Total delay | 52 (22,112) | 62 (23, 128) | 55 (47-62) |

TB – tuberculosis; SAMVAD – sensitization and advocacy in marginalised and vulnerable areas of the district (an active case finding strategy); HCP – health care provider; IQR – interquartile range

*delay was calculated from symptom onset whereas in our study it was from the date of eligibility for sputum examination. All TB patients (irrespective of whether detected by PCF or ACF)
